# Lycopene: an antioxidant product reducing dithane toxicity in *Allium* *cepa* L.

**DOI:** 10.1038/s41598-023-29481-4

**Published:** 2023-02-09

**Authors:** Oksal Macar, Tuğçe Kalefetoğlu Macar, Kültiğin Çavuşoğlu, Emine Yalçın, Kürşad Yapar

**Affiliations:** 1 Department of Food Technology, Şebinkarahisar School of Applied Sciences, Giresun University, Giresun, Turkey; 2 Department of Biology, Faculty of Science and Art, Giresun University, Giresun, Turkey; 3 Department of Pharmacology, Faculty of Medicine, Giresun University, 28049 Giresun, Turkey

**Keywords:** Plant genetics, DNA damage and repair, Biochemistry

## Abstract

The current study was undertaken to assess the attenuating potential of lycopene against Dithane toxicity in *Allium* *cepa* L. roots. *A.* *cepa* bulbs were arranged in 6 groups. The control group was treated with tap water while the other groups were treated with 215 mg/L lycopene, 430 mg/L lycopene, 500 mg/L Dithane, 500 mg/L Dithane + 215 mg/L lycopene and 500 mg/L Dithane + 430 mg/L lycopene, respectively. When the treatments were completed, growth inhibition, biochemical, genotoxicity and meristematic cell injury analyses were performed. Lycopene did not cause any toxic effect when applied alone. While rooting percentage, root elongation, weight gain and mitotic index (MI) decreased in response to Dithane exposure, the frequency of micronucleus (MN) and chromosomal abnormalities (CAs) in addition to malondialdehyde (MDA) level and the catalytic activities of superoxide dismutase (SOD) and catalase (CAT) increased. Dithane promoted fragment, sticky chromosome, vagrant chromosome, unequal distribution of chromatin, bridge, nucleus bud and reverse polarization formation in meristem cells. Dithane also provoked meristematic cell injuries, including indistinct appearance of vascular tissue, epidermis cell damage and flattened cell nucleus. Lycopene mitigated all damage types, depending on the lycopene dose applied with Dithane. Hence, the data analysis revealed that lycopene provides exceptional antioxidant protection against the fungicide Dithane, which has devastating toxic potential.

## Introduction

More than 120,000 species of fungi are known today, and this number is increasing with thousands of new species discovered each year. Although only 10% of these species are plant pathogens, plant diseases caused by phytopathogenic fungi account for the greatest yield and crop losses and economic damage in agriculture and forestry^1^. Due to the acceleration in global population growth, it is foreseen that the increase in agricultural products should reach 50% by the 2050s^2^. The intensive use of various pesticides, including synthetic fungicides, is one of the most common options to maintain the yield and quality of crops. Fungicides are used primarily for fruits and vegetables and account for more than 35% of the global pesticide market share^3^. Despite the effectiveness of fungicides in eliminating fungal attacks from seeds and plants, the frequent application of these chemicals poses various risks such as disruption of the environment, stimulated resistance, hazards to resident organisms and noxious residuals^4^. Genetic structures of organisms encountered with fungicides may also change because of the mutagenic and carcinogenic potential of these chemicals^5^.

Dithane, a commercial formulation containing 80% Mancozeb, is a broad-spectrum contact fungicide that is utilized to combat fungal diseases in vegetables, fruits, crops and paddy fields^6^. Mancozeb, one of the ethylene bis (dithiocarbamates) type fungicides, was first introduced to the world market in the 1960s, and its use has been increasing in the 2020s because of its non-selective fungicidal action and inexpensiveness^7^. The antifungal action of Mancozeb is due to its power to inhibit the sulfhydryl groups of both the amino acids and enzymes in the cells of fungal organisms, leading to the breakdown of respiration, lipid metabolism and ATP production^8^. Some adverse effects depending on Mancozeb exposure were reported as visceral diseases, nervous system disorders, skin damage, hormonal dysfunctions, reproductive system abnormalities and genetic defects^6^. Monitoring studies on Ethylene revealed that Ethylenethiourea, the most active degradation product of Dithane, is the main crucial toxicological risk factor^9^. Although the scientific area is replete with papers reporting the genotoxic capacity of fungicides on various organisms, multifaceted studies evaluating metabolic and chromosomal irregularities on plants caused by Dithane fungicide are limited^10^.

The pigment lycopene (molecular formula: C_40_H_56_) was first identified by Millardet in 1876 in tomato fruit with the name "soanorubin", and then purely isolated and named "lycopene" by Schunk in 1903^11^. As a naturally synthesized lipophilic carotenoid, it is also known as Ψ, Ψ-carotene and provides reddish hue to plants, such as tomatoes, watermelon, pink grapefruit and papaya^12,13^. Since the human metabolism cannot produce lycopene, it must be obtained through diet. Once it obtained from foods rich in lycopene, it can be stored in different parts of the body, including adrenals, prostate, liver, skin and brain^14^. One of the most remarkable advantages of a lycopene-rich diet is that lycopene is a natural phytonutrient that can be consumed in high amounts as a dietary supplement without harming health^15^. As a nutraceutical, lycopene has important applications against various ailments such as melanoma, cancer, infertility, obesity, cardiovascular or inflammatory diseases, oxidative stress-related metabolic failures and neurobehavioral disorders^12^. Among the 600 known natural carotenoids, including carotene and tocopherol, lycopene has been referred to as the most potent radical scavenger in vitro. It is endowed with impressive features to elevate the catalytic activities of enzymatic antioxidants and the amounts of non-enzymatic antioxidants to protect DNA and other macromolecules^16^.

The present study focused on the alleviating potential of lycopene against Dithane-induced toxicity in *A.* *cepa*, a widely employed model organism, to investigate the cytotoxic and genotoxic effects of harmful contaminants. The Allium test is also correlated with various test systems. This test is indispensable for distinguishing environmental pollution. Its results can provide a basis for the use of other test systems. Roots are the most sensitive and reliable system for studying the interaction mechanism of pesticides as the first place in which chemicals act^17^. As a result of its success and reliability, many researchers have studied the adverse effects of pesticides on *A.* *cepa*^18–20^.

In order to eliminate the lack of data available in the literature on this subject, some growth inhibition, biochemical, genotoxicity and meristematic cell injury analyses were performed in *A.* *cepa* roots exposed to Dithane fungicide and different doses of lycopene.

## Materials and methods

### Procedure for preparation of test solutions and *A. cepa* bulbs

Healthy bulbs of *A.* *cepa* (2n = 16) were purchased from a local market in Giresun province. The bulbs were cultivated without any use of pesticides or hormones. The initial weights of the bulbs used in the experiment varied between 9.48 g and 9.56 g. The dry scales of the bulbs were peeled off and the old roots were removed from the basal plate. Dithane M45 Special containing 80% Mancozeb was obtained from Dow AgroSciences (Istanbul/Turkey) to prepare the aqueous test solutions. Lycopene extracted from *Solanum* *lycopersicum* was purchased from Sepe Natural Organic Products (Izmir/Turkey). Experimental doses of the solutions were determined by considering the previous studies by Üstündağ et al.^21^ for Dithane and Çavuşoğlu et al.^22^ for lycopene. Basal plates of the bulbs in the control group were kept in contact with tap water.

**Figure 1:**
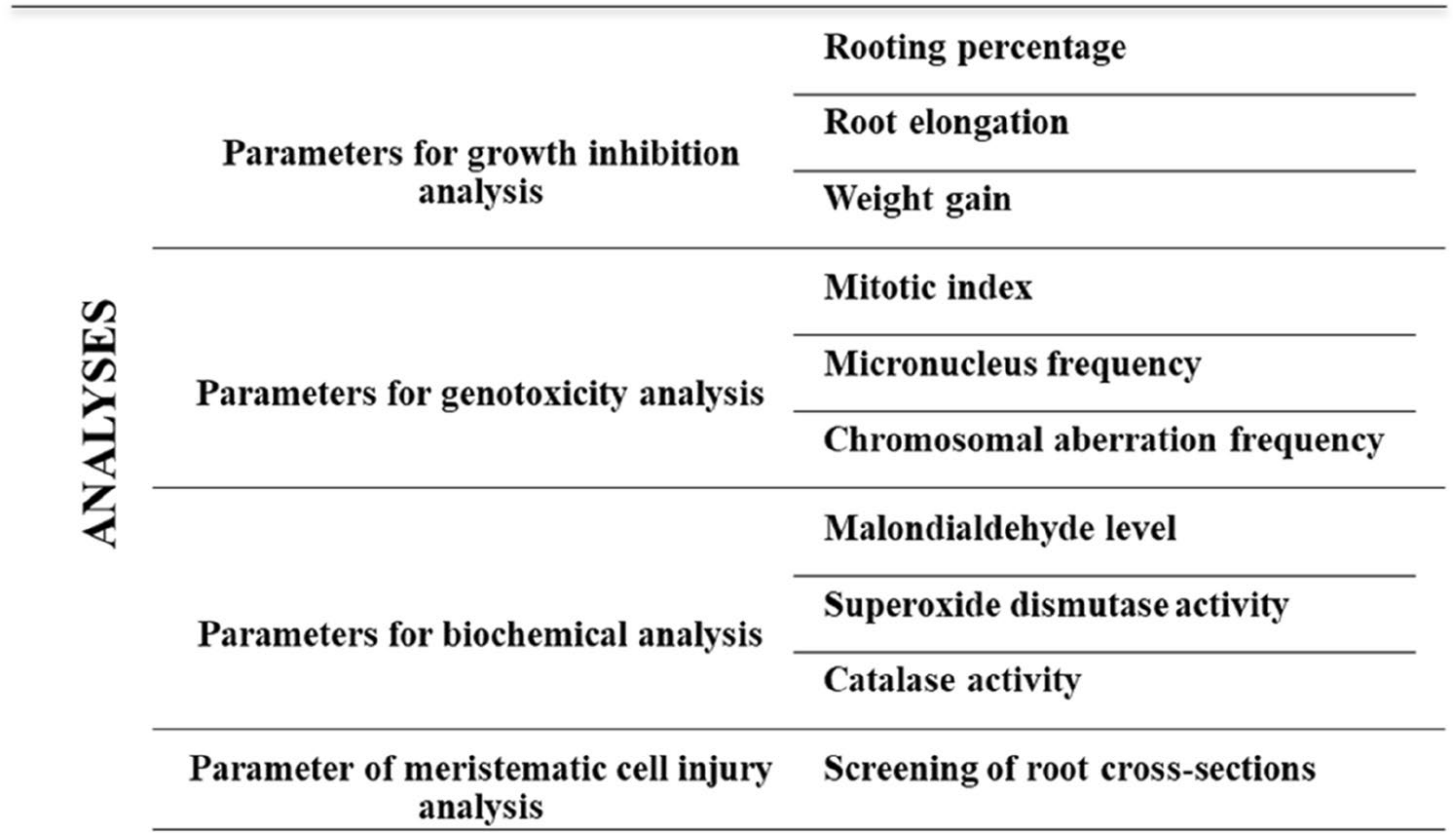
Analysis-parameter relationship in the study design.

On the other hand, the bulbs in the other groups were treated in the same way as the control, with 215 mg/L lycopene (Ly1), 430 mg/L lycopene (Ly2), 500 mg/L Dithane (D), 500 mg/L Dithane + 215 mg/L lycopene (DLy1) and 500 mg/L Dithane + 430 mg/L lycopene (DLy2), respectively. The solutions in contact with the bulbs were refreshed daily to maintain constant concentrations. The use of plants and the experiments in the present study complies with the relevant international, national and institutional guidelines. The application procedure was maintained for 72 h under stable laboratory conditions. A diagram reflecting the analysis-parameter relationship is presented below (Fig. 1). For rooting percentage and weight gain, 50 bulbs of each group were used. For root elongation and other analyses, 10 bulbs were used in each group.

### Evaluation of growth inhibition

Rooting percentage, the first parameter to determine the influences of Dithane and lycopene solutions on the growth of *A.* *cepa*, was calculated using the slightly modified formula of Atik et al.^23^ Eq. (1).

1 $${\text{Rooting percentage }}\left( \% \right) \, = \, \left( {\text{number of bulbs with roots / total number of bulbs}} \right) \, \times \, 100.$$

To assess root elongation levels, the length of newly emerged adventitious roots (cm) was measured with a ruler from the basal plate to the tip of each root.

In order to evaluate the total weight gain at the end of the rooting period, the differences between the pre- and post-treatment weights of the bulbs were calculated.

### Evaluation of genotoxicity

The genotoxic effects of the test solutions were found out using the *A.* *cepa* biomonitoring test, which reveals the MI and the intensity of CAs and MN. At the end of the 72 h experimental duration, genotoxicity examination slides were prepared from decapitated *A.* *cepa* roots according to the method of Staykova et al.^24^. The classical squash preparation technique was utilized for root tips stained with acetocarmine dye (1%). MI value and the incidence of MN and CAs were calculated by screening the same slides under a research microscope (Irmeco IM-450 TI) at 400× magnification. MI value was considered the ratio of cells in the mitotic stage of the total cells observed. A total of 10,000 cells (1000 cells from 10 root tip slides) and a total of 1000 cells (100 cells from 10 root tip slides) were monitored for each group to evaluate MI and CAs (including MN), respectively.

### Evaluation of biochemical responses

SOD (EC 1.15.1.1) and CAT (1.11.1.6) activities were assayed spectrophotometrically following the procedures developed by Beauchamp and Fridovich^25^ and Zhang et al.^26^, respectively. The supernatant containing the enzymes was extracted from *Allium* root tissues by the same procedure proposed by Zou et al.^27^. Enzymatic inhibition of photoreduction of nitroblue tetrazolium was followed at a wavelength of 560 nm to determine SOD activity. Enzymatic removal of the hydrogen peroxide (H_2_O_2_) substrate was monitored at a wavelength of 240 nm for the estimation of CAT activity. The units of SOD and CAT activities were presented as U/mg FW and OD_240_nm/min.g FW, respectively.

One of the most distinct bioindicators under stressful conditions inducing oxidative imbalance in the cells is membrane damage, manifested by MDA accumulation. MDA increment resulting from the exposure of the bulbs to test solutions was determined spectrophotometrically following the procedure developed by Heath and Packer^28^. Derivatized MDA abundances of the samples were monitored at wavelengths of 532 nm and 600 nm. The unit of MDA level was presented as µM/g FW. All procedures for biochemical parameters were repeated three times from beginning to end.

### Evaluation of meristematic cell injury

The influences of the test solutions on the meristem tissue of the samples were investigated in the root tips. Cross-sections of the roots were taken manually from fresh root tips with a razor blade. Methylene blue (2%) was used to stain tissues placed on slides observed and photographed at 200× magnification under a research microscope (Irmeco IM-450 TI). Tissues were graded according to their damage severity in four categories as “uninjured”, “slightly injured”, “moderately injured” and “extremely injured”.

### Statistical analysis

Statistical analyses were performed with the help of SPSS 22 analytical software for Windows. Differences between the results (presented as mean ± standard deviation in tables) were analyzed at a significance level of p < 0.05 using One-way ANOVA followed by Duncan’s test.

## Results and discussion

### Physiological findings

Table 1 shows the effects of Dithane and lycopene solutions on some growth parameters in *A.* *cepa*. The Ly1 and Ly2 groups, which were treated with two different doses of lycopene, showed 100% rooting success. The high rooting percentage in the control group was the most important criteria indicating the viability of the selected bulbs for the study. Root length and weight increase observed in lycopene-treated bulbs in the Ly1 and Ly2 groups were not statistically different from that of the control group. The unaffected growth of bulbs in the Ly1 and Ly2 groups indicated that the applied doses of lycopene were not toxic for *A.* *cepa*. On the other hand, the rooting percentage exhibited a parlous decrease in the bulbs exposed to Dithane (D group). In the same group, a 60% reduction in root length and 80% reduction in weight gain were observed, respectively, compared to the control. These significant inhibitions in growth pointed out that Dithane is a growth inhibitory agent for *A.* *cepa*. Our data was in full agreement with the results of Üstündağ et al.^21^ who noted the suppressing effects of Dithane on the growth of *A.* *cepa*. In addition, Buts et al.^29^ indicated that excessive concentrations of Dithane repressed the growth and development stages of *Abelmoschus* *esculentus*. Furthermore, Mancozeb, the active ingredient of Dithane, induced a time- and dose-dependent retardation in root elongation and the root number of *A.* *cepa*^19^. The rooting process is contingent on cell proliferation and the activation of enzymes that govern cell wall loosening and elongation during the differentiation phase^30^. Thus, the morphotoxic potential of Dithane on *A.* *cepa* may be due to either mito-suppression of cells or the deactivation of enzymes in growth by Mancozeb. In the DLy1 and DLy2 groups exposed to the lycopene–dithane mixtures, lycopene dose-dependently attenuated the toxicity of fungicide on growth in the bulbs. Rooting percentages of the DLy1 and DLy2 groups were 64% and 77%, respectively. In addition, the root length and weight increase of bulbs in these groups were significantly higher than in D group exposed only to Dithane. However, the growth restoration observed in the DLy1 and DLy2 groups did not reach control levels. The lycopene-linked limitation of growth inhibition induced by Spirodiclofen pesticide in *A.* *cepa* roots was previously demonstrated by Çavuşoğlu et al.^31^. This study is the first to demonstrate the reducing effect of lycopene against the growth-restricting toxicity of another pesticide, Dithane, in *A.* *cepa*. The protection provided by lycopene was attributed to the modulation potential of lycopene in oxidative stress through its antioxidant effect, free radical scavenging capacity and chelating power^32^.

**Table 1:** Influences on selected growth parameters in *A.* *cepa* exposed to dithane and lycopene.

Groups	Rooting percentage (%)	Root length (cm)	Weight increase (g)
Control	98	8.50 ± 3.24^a^	+ 5.50^a^ (9.51 ± 1.75–15.01 ± 2.68)
Ly1	100	8.60 ± 3.26^a^	+ 5.75^a^ (9.55 ± 1.76–15.30 ± 2.72)
Ly2	100	8.80 ± 3.32^a^	+ 5.82^a^ (9.53 ± 1.73–15.35 ± 2.74)
D	55	3.40 ± 0.78^d^	+ 1.00^d^ (9.56 ± 1.70–10.56 ± 1.83)
DLy1	64	4.50 ± 1.16^c^	+ 2.60^c^ (9.48 ± 1.68–12.08 ± 1.95)
DLy2	77	6.20 ± 1.54^b^	+ 4.10^b^ (9.52 ± 1.71–13.62 ± 2.16)

Control: tap water, Ly1: 215 mg/L lycopene, Ly2: 430 mg/L lycopene, D: 500 mg/L dithane, DLy1: 500 mg/L dithane + 215 mg/L lycopene, DLy2: 500 mg/L dithane + 430 mg/L lycopene. Lowercase letters in the same column indicate statistical significance between the means (p < 0.05). For rooting percentage and weight gain, 50 bulbs were used; for root elongation, ten bulbs were used in each group.

### Genotoxicity findings

#### MN frequency

**Figure 2:**
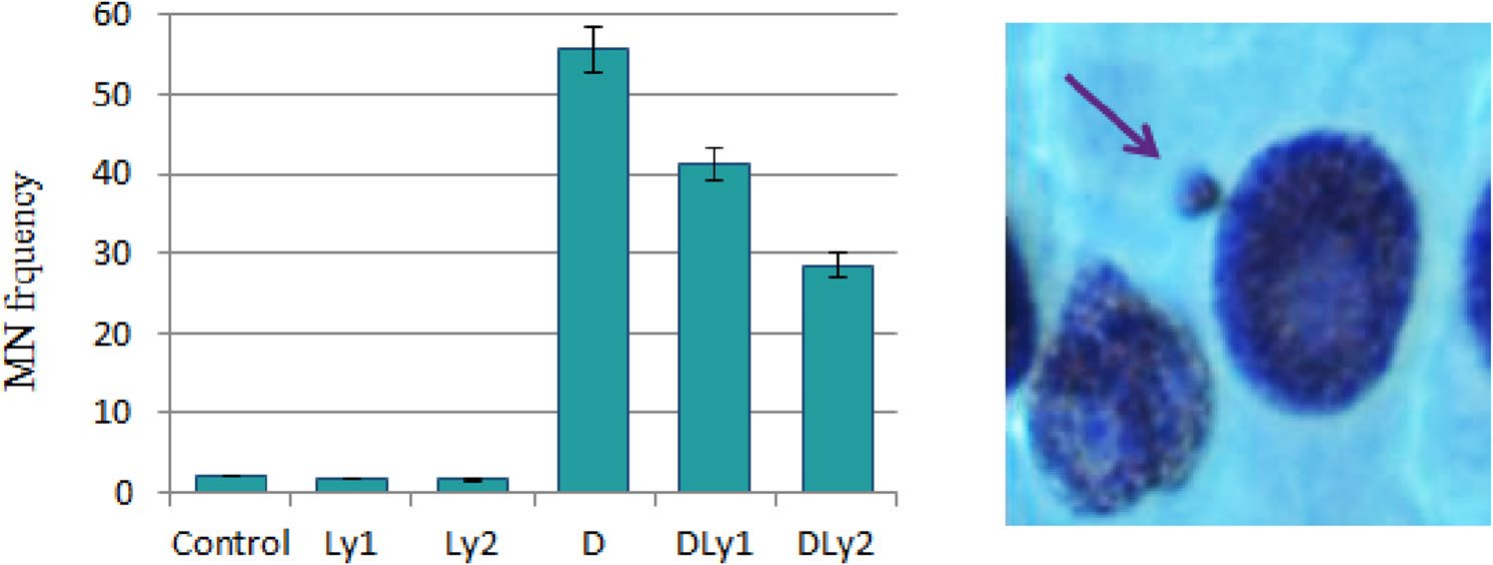
MN frequency and MN appearance induced by dithane. 1000 cells were scored for each group to evaluate MN frequency.

Figure 2 depicts the genotoxic impacts of Dithane on MN frequency and MN appearance on *A.* *cepa*. In vivo assessment of genotoxicity was performed using MI and scores of MN and CAs. MN is defined as a tiny extra nuclear body formed by the failure of a chromosome or chromosome fragment to be included in one of the daughter nuclei during mitosis^33^. Investigation of the presence of MN, which is a successful biomarker of mutagenicity and genotoxicity in cells, is a sensitive tool in detecting toxicity. Since MN is easily discernible in *A.* *cepa* cells, it reduces the use of experimental animals in the inspection of toxic chemicals^34^. Here, in our study, there was not a significant change in MN frequency in the Ly1 and Ly2 groups compared to the control. On the contrary, the treatment with Dithane in the group D induced a burst in MN formation (Fig. 2). Similar data on the MN stimulating effect of Dithane in the meristematic cells of *A.* *cepa* roots were reported by other researchers^21,35^. Pesticides considered to be “clastogenicity triggers” induce MN formation^36^. As our study confirmed, Asita and Makhalemele^9^ suggested that Dithane is a conspicuously genotoxic substance for *A.* *cepa*.

#### Mitotic index

**Figure 3:**
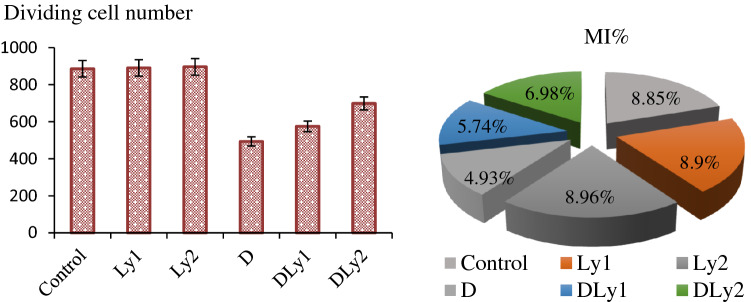
The effects of dithane and lycopene treatments on dividing cell numbers and MI (%). 10,000 cells were scored for each group to evaluate for MI.

MI, one of the parameters contributing to a clearer understanding of plant growth, has been used to determine the root growth rate resulting from cell proliferation^37^. As the differences between MI values were not significant in the first three groups (Control, Ly1 and Ly2) (Fig. 3), this study revealed that lycopene was not anti-proliferative in *A.* *cepa* cells. In contrast to the groups treated with lycopene solutions, group (D) exposed to Dithane exhibited a dramatic decrease in MI. Bianchi et al.^38^ noted that MI is a strong indicator in determining the cytotoxicity of a pollutant. Considering that there is a direct relationship between the MI and mitosis rate^39^, according to the results of this study, Dithane was an inhibitor of cell division in *Allium* roots. The results of the MI assay used in this study were in line with the previous studies indicating the mitosuppressive effects of Dithane or Mancozeb^5,19,21,40,41^. Singh et al.^40^ suggested that the mechanisms underlying the decrease in MI could be a blockage in the G1 phase that directly suppresses DNA synthesis or a limitation in the G2 stage that stops cells from initiating mitosis.

#### CAs frequencies

**Figure 4:**
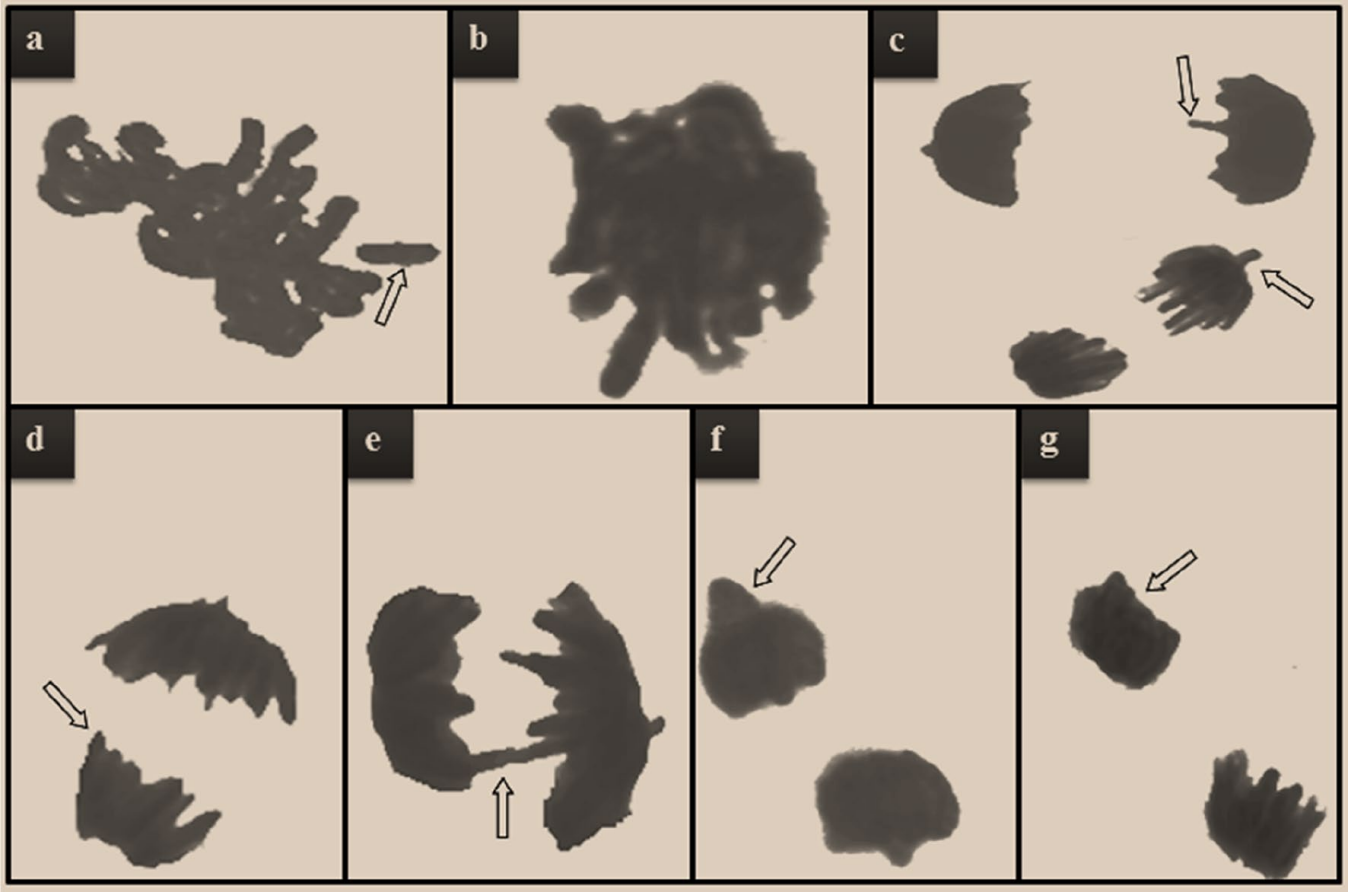
Chromosomal aberration types in *A.* *cepa* root cells exposed to dithane. Chromosomal fragment (**a**), sticky chromosome (**b**), vagrant chromosome (**c**), unequally distributed chromatins (**d**), chromosomal bridge (**e**), nucleus bud (**f**), reverse polarization (**g**).

Examining CAs in *A.* *cepa* root tip cells is crucial in demonstrating the presence of clastogenic, genotoxic, or aneugenic agents in the environment. Although small amounts of CAs can occur spontaneously in cells, exposure to chemical or physical contaminants increases the frequency of these abnormalities depending on the severity of the stimulus. Similar to the MI and MN results, exposure to different doses of lycopene solutions in the bulbs of the Ly1 and Ly2 groups did not induce a significant change in CA formation compared to the control group (Table 2). On the other hand, bulbs in the D group exposed to Dithane solution showed notable increases in various types of CAs (Table 2, Fig. 4). Chromosomal fragments (Table 2, Fig. 4a) and stickiness (Table 2, Fig. 4b) were the two most common CAs types. Bolsunovsky et al.^42^ suggested that fragment formation was the main source of MN accumulation in stressed *Allium* cells. According to El-Ghamery et al.^43^ stated that sticky chromosomes lead to chromosomal fragmentation and bridge formation. Stickiness is a chromatid-type disorder resulting from chromosomal entanglement and depolymerization, condensation or fragmentation of DNA^19^. Pollutants that often induce irreversible sticky chromosomes are so toxic that they can even cause cell death^44^. In the D group, an increase was detected in the abundance of vagrant chromosomes (Table 2, Fig. 4c), an indicator of aneuploidy risk^18^. Since the formation of vagrant chromosomes causes unequal segregation of chromosomes, it eventually results in unequally sized or irregularly shaped nuclei in daughter cells^45^. Other chromosomal abnormalities detected in Dithane-administered bulbs were listed as unequally distributed chromatins (Table 2, Fig. 4d), chromosomal bridge (Table 2, Fig. 4e), nucleus bud (Table 2, Fig. 4f) and reverse polarization (Table 2, Fig. 4g) in order of abundance. Bridges arising from the chromosome or chromatid ruptures and fusion can cause structural chromosome mutation^43^, while spindle organization-disarrangement is a fundamental factor that leads to vagrant chromosome accumulation and reverses polarization^46^. Additionally, increased oxidative imbalance in the cell is often cited as the cause of nuclear damage^47^. Several researchers have uttered pesticide-related decreases in MI along with increases in CAs and MN abundance in plant cells, including *A.* *cepa*^48,49^. Maity and Maitra^5^ indicated that Dithane provoked an elevation in CAs such as fragmentation, condensed/granulated chromatin, bridge, c-metaphase and lagging chromosomes in *Cicer* *arietinum* root cells. Moreover, the study designed by Maity^41^ indicated that chromosomal bridges, lagging early anaphase, c-metaphase and granulated chromatin were among the Dithane-induced CAs in *Vigna* *mungo* L. root tips. In another study using *A.* *cepa* as a model, Dithane caused an increase in CAs, such as fragmentation, bridge formation, and sticky chromosomes, while decreasing MI^50^. The common feature of all these studies, including ours, was that they attested that Dithane was genotoxic. The genotoxic potential of Dithane was attenuated by lycopene in the DLy1 and DLy2 groups (Table 2). The dose-dependent curative power of lycopene applied in a mixture with Dithane continued in genotoxicity and growth parameters without exception. The higher the dose of lycopene mixed with Dithane, the greater the increase in MI value and the reduction in MN and CAs levels. The protective or ameliorative potential of lycopene against the toxic effects of pesticides such as Chlorpyrifos, Diazinon, Malathion, Deltamethrin, Rotenone and Cypermethrin was previously reported by Hedayati et al.^32^. In addition, Çavuşoğlu et al.^22^ demonstrated the ability of lycopene to mitigate the genotoxic damage of another stress factor, UV-C, in *A.* *cepa* root cells. In the study mentioned above, lycopene increased MI while decreasing the levels of MN and CAs, as in the results of our study. In addition, Aslanturk and Celik^51^ proved with the *A.* *cepa* test system that lycopene reduces CAs triggered by Ethyl methanesulfonate, which is known to be a mutagen. The present study provides the first evidence showing the anti-genotoxic property of lycopene against the pesticide Dithane. Lycopene protects vital biomolecules such as DNA by scavenging reactive oxygen species (ROS) and regulating the detoxification apparatus, thereby preventing cancer and mutagenicity^52^. Indeed, the antioxidant activity of lycopene was evidenced by the remarkable suppressive role of lycopene in the mutagenic and genotoxic influences of the H_2_O_2_ radical^53^.

**Table 2:** Mitigative role of lycopene against genotoxicity induced by dithane.

Damages	Control	Ly1	Ly2	D	DLy1	DLy2
CFR	0.00 ± 0.00^d^	0.00 ± 0.00^d^	0.00 ± 0.00^d^	45.70 ± 4.56^a^	37.20 ± 3.58^b^	26.10 ± 2.14^c^
SC	1.30 ± 0.84^d^	0.98 ± 0.56^d^	0.36 ± 0.42^d^	35.30 ± 3.87^a^	27.10 ± 2.98^b^	20.50 ± 2.52^c^
VC	0.00 ± 0.00^d^	0.00 ± 0.00^d^	0.00 ± 0.00^d^	28.30 ± 2.85^a^	21.50 ± 2.36^b^	15.30 ± 1.94^c^
UDC	0.00 ± 0.00^d^	0.00 ± 0.00^d^	0.00 ± 0.00^d^	18.90 ± 1.75^a^	12.60 ± 1.34^b^	8.60 ± 1.12^c^
CB	0.16 ± 0.34^d^	0.00 ± 0.00^d^	0.00 ± 0.00^d^	15.60 ± 1.68^a^	10.40 ± 1.53^b^	6.70 ± 1.16^c^
NB	0.00 ± 0.00^d^	0.00 ± 0.00^d^	0.00 ± 0.00^d^	10.50 ± 1.18^a^	6.10 ± 0.86^b^	2.50 ± 0.64^c^
RP	0.00 ± 0.00^d^	0.00 ± 0.00^d^	0.00 ± 0.00^d^	6.40 ± 0.96^a^	3.70 ± 0.62^b^	1.50 ± 0.41^c^

Control: tap water, Ly1: 215 mg/L lycopene, Ly2: 430 mg/L lycopene, D: 500 mg/L dithane, DLy1: 500 mg/L dithane + 215 mg/L lycopene, DLy2: 500 mg/L dithane + 430 mg/L lycopene. Lower case letters in the same line indicate statistical significance between the means (p < 0.05).

*CFR* chromosomal fragment, *SC* sticky chromosome, *VC* vagrant chromosome, *UDC* unequally distributed chromatins, *CB* chromosomal bridge, *NB* nucleus bud, *RP* reverse polarization.

#### Biochemical findings

MDA content and antioxidant enzyme activities were used as markers of biochemical responses to Dithane and lycopene in root tip cells of *A.* *cepa* (Table 3). Oxidative stress is one of the main causative circumstances of cell disturbances induced by toxic substances. Since plants are sessile organisms, they face many environmental challenges. Under normal conditions, various free radicals formed due to these challenges can be easily defeated by the internal defense mechanisms in the cells. However, if the plants cannot evade these stressors, also called reactive oxygen species (ROS), depending on the intensity or exposure time of the stress factor, a situation called “oxidative stress” occurs. MDA is a tiny organic compound usually formed in the case of lipid peroxidation in biological membranes due to the destructive nature of ROS^54^. No significant difference was observed in the MDA content of bulbs treated with lycopene (Ly1 and Ly2), both compared to each other and to the control (Table 3). On the other hand, the MDA level in the group treated with Dithane (D) was approximately 2.2 times that of the control group. Similar to MDA levels, there was no significant difference between the SOD and CAT enzyme activities of the first three groups (Ly1, Ly2 and D). However, SOD and CAT activities of bulbs in the D group were approximately 2.4 and 2.6 times that of the control, respectively. SOD and CAT are two antioxidant enzymes with the catalytic activity that deplete superoxide and H_2_O_2_ radicals in cells^55^. Considering the high catalytic activities of SOD and CAT enzymes and MDA accumulation, this study showed that Dithane elicited by oxidative stress in *A.* *cepa* root cells for the first time. However, it was reported that Ridomil pesticide containing mancozeb induced high levels of MDA and SOD and CAT activities in *A.* *cepa* root meristem cells^56^. According to Saber and El-Aziz^57^, owing to its chemical structure containing transition metals, Dithane (Mancozeb) causes oxidative stress by promoting the production of ROS via the Fenton reaction. As Mahapatra et al.^58^ mentioned, high amounts of ROS accumulated in plant cells exposed to pesticides initiate oxidative and genotoxic stress responses and cause cyto-genotoxicity at a level that damages DNA. In the DLy1 and DLy2 groups exposed to the lycopene–dithane mixtures, lycopene dose-dependently lessened the oxidative stress in the bulbs, considering the declined MDA abundance and decelerated SOD and CAT activities (Table 3). Although many publications are investigating the relationship between lycopene and fluctuation in oxidative stress biomarkers resulting from pesticide administration^59,60^, our results were consistent with the data of Çavuşoğlu et al.^31^, which showed the protective potential of lycopene against pesticide-related oxidative stress in *Allium*. Scolastici et al.^52^ stated that antioxidant molecules including lycopene must remove ROS through reacting with them to convert these radicals into harmless molecules or by interrupting the chain reactions initiated by ROS. For instance, lycopene is widely accepted as the strongest singlet oxygen scavenger among the naturally synthesized carotenoids due to the large number of dienes in the structure of this pigment^61^. As a large ROS quencher, lycopene ceases peroxidation in the lipid bilayer of membranes and protects the genetic material of cells^53^. Its lipophilic nature allows lycopene to act specifically on biological membranes and near membrane components^62^.

**Table 3:** Biochemical responses to Dithane and lycopene in root tip cells of *A.* *cepa.*

Groups	MDA (µM/g FW)	SOD (U/mg FW)	CAT (OD_240 nm_/min.g FW)
Control	16.60 ± 3.58^d^	140.20 ± 06.86^d^	1.35 ± 0.48^d^
Ly1	15.90 ± 3.45^d^	135.30 ± 06.52^d^	1.30 ± 0.38^d^
Ly2	14.70 ± 3.26^d^	132.80 ± 06.24^d^	1.27 ± 0.32^d^
D	36.30 ± 5.38^a^	330.40 ± 12.65^a^	3.54 ± 0.86^a^
DLy1	31.40 ± 4.98^b^	260.50 ± 10.96^b^	2.75 ± 0.66^b^
DLy2	25.60 ± 4.24^c^	200.80 ± 08.84^c^	2.28 ± 0.52^c^

Control: tap water, Ly1: 215 mg/L lycopene, Ly2: 430 mg/L lycopene, D: 500 mg/L dithane, DLy1: 500 mg/L dithane + 215 mg/L lycopene, DLy2: 500 mg/L dithane + 430 mg/L lycopene. Lower case letters in the same column indicate statistical significance between the means (p < 0.05).

#### Anatomical alterations

**Figure 5:**
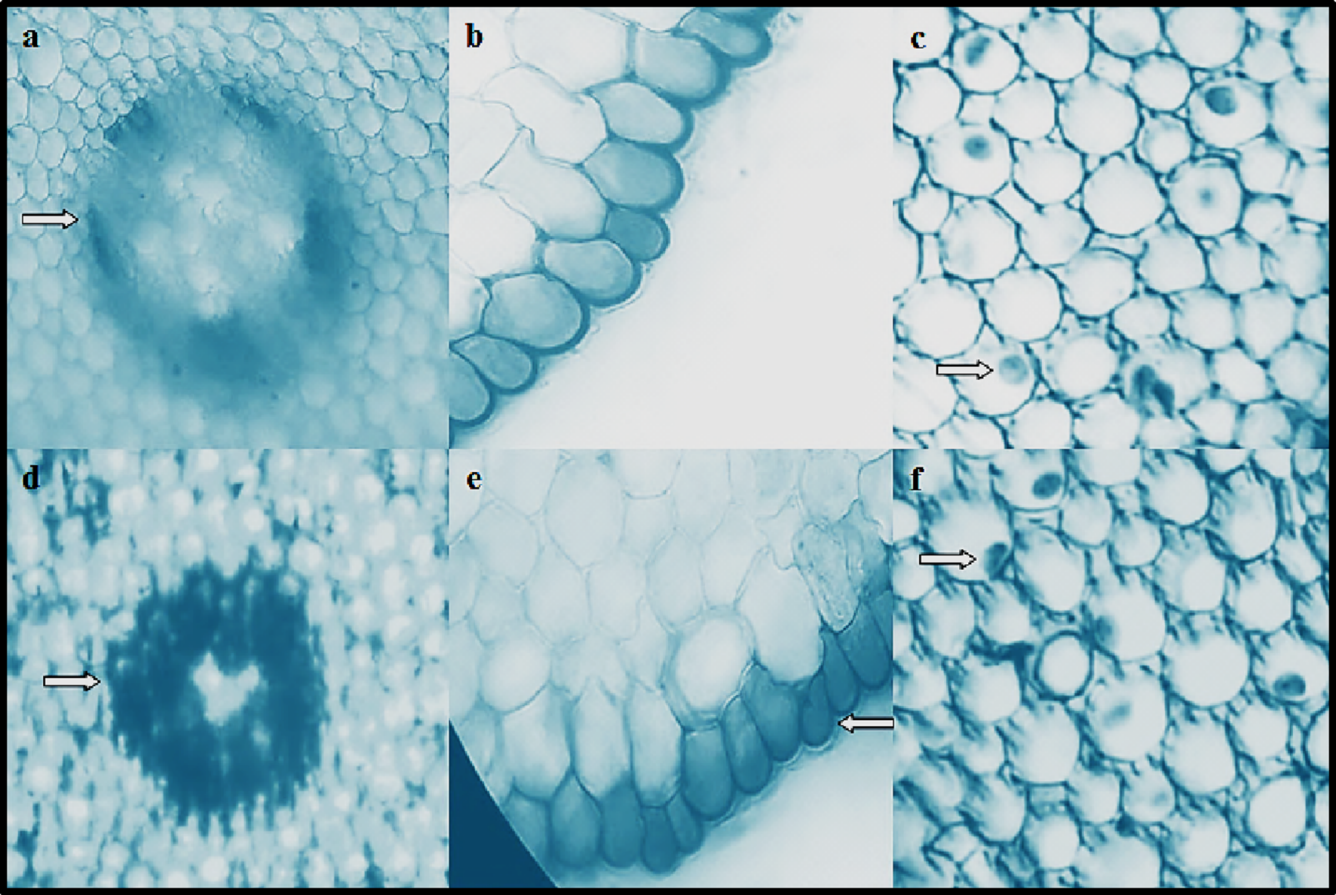
Meristematic cell injuries provoked by dithane exposure. Normal appearance of vascular tissue (**a**), normal appearance of epidermis cells (**b**), normal appearance of cell nucleus-oval (**c**), indistinct appearance of vascular tissue (**d**), epidermis cell damage (**e**), flattened cell nucleus (**f**).

A cross-section of root tips was utilized to analyze the alleviating power of lycopene against meristematic cell injuries triggered by Dithane. Like the control group, lycopene did not cause any meristematic cell injury regardless of the dose used in the Ly1 and Ly2 groups (Table 4, Fig. 5a–c). On the other hand, various damages, including indistinct appearance of vascular tissue (Table 4, Fig. 5d), epidermis cell damage (Table 4, Fig. 5e) and flattened cell nucleus (Table 4, Fig. 5f) appeared in the cells located in the meristematic zone of the roots exposed to Dithane application. The defects observed in the D group were rated as "moderately injured" for the indistinct appearance of vascular tissue and "extremely injured" for epidermis cell damage and flattened cell nucleus. When lycopene was co-administered with Dithane, all damage types were reduced depending on the dose of lycopene in the mixture (DLy1 and DLy2). Indeed, the indistinct appearance of vascular tissue and flattened cell nucleus damages were not observed in the Dly2 group that showed the best recovery. Although studies are unveiling the devastating effects of pesticides on the root anatomy of plants^63^, this study is the first to demonstrate root meristematic damage induced by Dithane in *A.* *cepa*. Damage to the vascular tissue responsible for transporting nutrients and water to the upper parts of the plant is fatal enough to affect plant survival. Disorders in the vascular tissue integrity clearly show the negative effect of excessive oxidative stress caused by Dithane exposure on cell membranes and is also one of the main reasons for the growth inhibition observed in the study. The root epidermis is the first layer where the roots encounter harmful chemicals in the growth medium. In this case, the tightening that appears in the cells of this tissue is both an injury and an effort to prevent the chemical compound from entering the inner tissues. Yalçın et al.^64^ suggested that a flattened cell nucleus may result from direct damage to the genetic material caused by the genotoxic chemical. Although the potential of lycopene to protect *A.* *cepa* root meristem cells against harmful factors has been previously shown by Çavuşoğlu et al.^22^, there is no other study in the literature showing that it reduces root anatomy disorders caused by Dithane.

**Table 4:** Alleviating role of lycopene against meristematic cell injuries induced by dithane.

Groups	IAVT	ECD	FCN
Control	**−**	**−**	**−**
Ly1	**−**	**−**	**−**
Ly2	**−**	**−**	**−**
D	**++ **	** +++ **	** +++ **
DLy1	** + **	** ++ **	** + **
DLy2	**–**	** + **	**–**

Control: tap water, Ly1: 215 mg/L lycopene, Ly2: 430 mg/L lycopene, D: 500 mg/L dithane, DLy1: 500 mg/L dithane + 215 mg/L lycopene, DLy2: 500 mg/L dithane + 430 mg/L lycopene.

*IAVT* indistinct appearance of vascular tissue, *ECD* epidermis cell damage, *FCN* flattened cell nucleus. (−): un-injured, ( +): slightly injured, (++): moderately injured, (+++): extremely injured.

#### Toxicity reducing role of lycopene

The results of growth inhibition, biochemical, genotoxicity and cell damage parameters obtained from this study clearly show dithane-induced toxicity and the reducing effect of lycopene against this toxicity in *A.* *cepa.* The use of plant extracts to lessen the toxicity of environmental pollutants, such as heavy metals and pesticides, has increased over the years^65^. Due to having the highest antioxidant capacity of all the carotenoid pigments lycopene gains the uttermost attention. Lycopene protects vital cell structures such as DNA and cell membranes against ROS and free radicals caused by dithane with its notable scavenging and chelating properties. In addition, being the most potent singlet oxygen and peroxyl radical scavenger among carotenoids due to its high diene number also contributed to its protective effect ^61,66^. It has been emphasized in other studies that lycopene has a protective effect against genotoxic and toxic substances such as UV, heavy metals and pesticides^22,31,32,51,65,67^.

## Conclusion

In conclusion, the results proved the toxicity of Dithane fungicide and the protective role of lycopene in a comprehensive in-vivo study. Exposure to Dithane inhibited growth, triggered genotoxicity, initiated oxidative stress and led to various anatomical defects in the integrity of meristematic cells. Co-administration of lycopene with Dithane dose-dependently reduced the harmful effects of the fungicide in all parameters examined. The notable restoration of oxidative stress indicators following the addition of lycopene to Dithane solution points out the antioxidant nature of the pigment. The suitability of A. cepa has been confirmed to demonstrate the therapeutic potential of lycopene, a naturally synthesized “reddish guard”, against the harms of a toxic agent. Due to its extraordinary qualities, lycopene should not be neglected in daily nutrition and the shielding effects of lycopene against other risky toxicants should be inquired about in more detail.

## Data Availability

All data generated or analysed during this study are included in this published article [and its supplementary information files].
